# Algicidal Characteristics of *Bacillus cereus* Strain PT1 Against *Microcystis aeruginosa* in Sulfate-Type Saline–Alkaline Environments

**DOI:** 10.3390/microorganisms14030647

**Published:** 2026-03-13

**Authors:** Qing Wang, Yucheng Cao, Yunna Xu, Keng Yang, Chuangwen Xu, Guoliang Wen, Jinfan Liu, Jianshe Zhang, Xiaojuan Hu

**Affiliations:** 1 National Engineering Research Center for Marine Aquaculture, Zhejiang Ocean University, Zhoushan 316022, China; wq1398903367@163.com (Q.W.); guowen66@163.com (G.W.); zhangjianshe@zjou.edu.cn (J.Z.); 2 South China Sea Fisheries Research Institute, Chinese Academy of Fishery Sciences/Southern Marine Science and Engineering Guangdong Laboratory (Zhuhai), Guangzhou 510300, China; cyc_169@163.com (Y.C.); 13265156173@163.com (Y.X.); yangkeng66@163.com (K.Y.); xuchuangwen1984@sina.com (C.X.); fj6abc@163.com (J.L.); 3 Sanya Tropical Fisheries Research Institute, Sanya 572018, China

**Keywords:** algicidal bacteria, metabolites, saline–alkali environment

## Abstract

Biologically controlling *Microcystis aeruginosa* blooms in saline–alkaline environments remains a major challenge in aquatic ecosystem management. Here, the algicidal performance of an indigenous algicidal bacterium, *Bacillus cereus* strain PT1 isolated from a sulfate-type saline–alkaline pond, against *M. aeruginosa* was evaluated, and the underlying metabolic mechanisms were elucidated using non-targeted metabolomics. PT1 exhibited pronounced, stable algicidal activity under saline–alkaline conditions, decreasing the algal cell density from 2 × 10^6^ to 1.25 ± 0.5 × 10^5^ cells mL^−1^ within 4 days at a rate of 93.75 ± 2.5% (*p* < 0.05). The above results demonstrate that strain PT1 has a significant lytic effect on *M. aeruginosa*. Non-targeted liquid chromatography–mass spectrometry analysis identified 298 PT1-induced accumulated metabolic features, and the top 30 candidates comprised organic acids and aromatic compounds, including benzoic acid, coumarin, malonic acid, and signaling-related molecules, including indoleacetaldehyde and nitroprusside. These differential metabolites were associated with algicidal-related pathways, including quorum sensing, two-component systems, ABC transporters, and tryptophan metabolism, outlining a coordinated “regulation–transport–metabolic remodeling” framework. Our findings demonstrate the potential of the indigenous algicidal strain PT1 from saline–alkali ponds to control *M. aeruginosa* blooms. They also provide an important theoretical basis and data foundation for further elucidating the molecular characteristics of algae solubilizing activity under saline–alkali conditions and developing microbial agents for preventing and controlling *Microcystis* blooms in saline–alkali ponds.

## 1. Introduction

China harbors extensive saline–alkali soils, estimated accounting for ~10.1% of the world’s saline–alkali land [1], which are predominantly concentrated in northern China, including major saline–alkali regions in Northeast China and the irrigated agroecosystems of Northwest China [2,3]; nevertheless, the large resource base of alkaline–saline environments remains only partially utilized for production because severe soil constraints and limited alkaline-tolerance improvement/management have long hindered effective agricultural use [4]. Currently, the development and utilization of saline–alkali water bodies remains very limited, with the overall utilization rate even below 2% [5]. The chemical compositions of saline–alkaline waters are complex and diverse, and they differ fundamentally from those of both freshwater and seawater systems. Their most prominent physicochemical features are high pH, carbonate alkalinity, and ionic strength [6]. Saline–alkaline waters are not simply “freshwater with more salts” or “diluted seawater”; they are typically carbonate-buffered (HCO_3_^−^/CO_3_^2−^-rich) systems dominated by Na^+^, sustaining persistently high pH and a distinct ionic milieu compared with both freshwater and seawater [7,8,9].

Such carbonate–alkaline chemistry can shift algae–bacteria coupling by strengthening or weakening EPS-mediated adhesion/aggregation at cell interfaces and by changing the stability and bioavailability of extracellular infochemicals (e.g., pH-sensitive quorum-sensing signals), thereby altering the relative contribution of contact-dependent versus diffusible-metabolite algicidal modes [8]. Therefore, the dissolution characteristics and mechanisms of algae in freshwater or seawater systems cannot be directly applied to saline–alkali water systems [8,10,11]. At present, how to scientifically and effectively utilize these potential water resources has become a key issue [12]. Furthermore, the unique “three-high” characteristics of saline–alkaline waters pose substantial challenges for precise water quality regulation in aquaculture systems [13].

Under the sustained stress from the typical “three-high” conditions of saline–alkaline ponds, *Microcystis* cyanobacteria exhibit strong environmental adaptability and gradually occupy a dominant ecological niche, becoming the prevailing algal group in such waters. During summers with elevated temperatures, cyanobacterial blooms dominated by *Microcystis aeruginosa* frequently occur [14]. Yu and Zepernick et al. [15,16] reported that *M. aeruginosa* is remarkably tolerant to highly alkaline environments and can maintain normal growth and metabolic activity, even under extreme alkaline conditions. Recent evidence further suggests that elevated pH in the alkaline range can favor Microcystis proliferation, with optimal growth reported near pH 9–10 under laboratory conditions [17], and alkaline salt stress (salt-alkalization) may promote *Microcystis* blooms and affect physiology and microcystin production [16].

In addition, Liu et al. [12] reported that saline–alkaline aquaculture ponds enriched in carbon and nitrogen are more likely to develop *Microcystis-* and *Pseudanabaena*-dominated cyanobacterial communities. Microcystins (MCs) produced by *Microcystis* can disrupt the balance of aquatic micro-ecosystems and induce acute or chronic physiological stress responses in cultured organisms, ultimately leading to reduced aquaculture yields and deteriorated product quality [18,19]. Moreover, high MC concentrations can accumulate throughout the food chain, posing risks of acute liver injury, gastroenteritis, and potential carcinogenic effects in humans and thereby threatening food safety [20,21]. Therefore, the development of efficient and environmentally friendly strategies for controlling *Microcystis* blooms in saline–alkaline ponds is of significant practical importance for the promotion of sustainable and high-quality saline–alkaline aquaculture development.

At present, strategies for controlling cyanobacterial blooms in aquatic systems are generally classified into physical, chemical, and biological techniques. Physical methods are difficult to implement effectively at large spatial scales, whereas chemical methods often suffer from poor selectivity, limited long-term effectiveness, and high secondary pollution risk, which is inconsistent with modern ecological aquaculture models [22]. In contrast, owing to their high efficiency and sustainability, biological approaches have become a major research focus in harmful algal bloom control. Microbial control strategies are the most promising technical route due to their precise mechanisms of action and strong environmental adaptability [23]. Algicidal bacteria, key functional groups in microbial algal control, can inhibit algal growth and reproduction through direct cell contact or indirect metabolic regulation; thus, they have become a focal point of research in cyanobacterial bloom mitigation.

Previous studies have successfully isolated various algicidal bacterial strains from different ecological niches, including water bodies, sediments, and soils, including *Bacillus, Alteromonas*, *Pseudomonas*, and *Streptomyces* species [24,25,26,27]. For example, Brookes et al. [28] isolated a *Streptomyces* strain capable of interfering with the normal growth of *M. aeruginosa* through the secretion of bioactive metabolites, whereas Ganf et al. [29] reported a distinct algicidal mode in which *Streptomyces* lysed algal cells through direct contact without secreting inhibitory substances. Lu et al. [30] demonstrated that benzoic acid produced by *Thalassospira* exerted a concentration-dependent inhibitory effect on *Karenia mikimotoi*. Additionally, extracellular polymers produced by *Bacillus amyloliquefaciens* DT exhibited excellent flocculation activity against *M. aeruginosa*, achieving a flocculation efficiency of 87.98% within 10 min [31]. *Paucibacter aquatile* DH15 has also been reported to possess multiple functional advantages, including direct algicidal activity, algicidal substance secretion, and MC degradation [32]. Although these studies provide solid theoretical and practical foundations for the application of algicidal bacteria, most investigations focused on freshwater or marine environments, and research targeting saline–alkaline waters remains relatively limited. Although extensive research has been conducted on algicidal bacteria, the research into saline–alkali water is still relatively limited, especially in the following aspects: (i) isolation, screening, and characterization/validation of native algicidal bacterial strains in saline–alkali systems; (ii) separation and identification of algicidal determinants (bioactive metabolites/enzymes/polymers); (iii) molecular mechanisms and algicidal mechanisms in saline–alkali systems based on direct/indirect algicidal patterns; (iv) efficacy verification and toxicological verification in actual production, including the degradation effects of algicidal substances and microcystin toxins [33,34,35,36,37,38].

Currently, studies on the algicidal effects of bacteria against *Microcystis* in saline–alkaline ponds and the underlying mechanisms remain scarce, and the associated molecular processes have not been systematically elucidated [39]. In this study, an indigenous bacterial strain, *Bacillus cereus* strain PT1 isolated from the microalgal environment of a saline–alkali pond in Helan County, Yinchuan City, Ningxia Hui Autonomous Region, was used as the experimental material, with *Microcystis* spp. as the target organism in saline–alkaline ponds. Strain PT1 was obtained by using continuous dilution plates in an alkaline–saline medium to isolate culturable bacteria from the sediment of saline–alkali ponds. Then, it was screened under saline–alkali conditions through microalgae co-culture experiments to identify the isolates with stable anti-microalgae activity. Strain PT1 exhibited strong algicidal performance under saline–alkali conditions, and thus was selected as a representative indigenous strain for further research. We conducted a systematic evaluation of its algicidal performance, employing non-targeted metabolomics to preliminarily characterize the algicidal properties of strain PT1 and identify potential active metabolites associated with its algicidal activity. The aim of this study is to provide a theoretical basis and data support for further elucidating the molecular characteristics of algae solubilizing activity under saline–alkali conditions and the development of microbial agents for controlling *Microcystis* in saline–alkali aquaculture systems.

## 2. Materials and Methods

### 2.1. Experimental Bacterial Strain

*B. cereus* PT1 was provided by the Innovation Team for Ecological Environment Regulation in Mariculture Ponds of the Chinese Academy of Fishery Sciences. It was isolated from the microalgal environment of a saline–alkali pond in Helan County, Yinchuan City, Ningxia Hui Autonomous Region. The original aquatic environment had a salinity of 0–2, pH of 7.8–9.3, and temperature of 26.5–29.5 °C.

### 2.2. Experimental Algal Species

The *M. aeruginosa* used in this study was sourced from the Innovative Team for Ecological Environment Regulation in Seawater Pond Culture of the Chinese Academy of Fishery Sciences. The microalgae were activated and proliferated using BG11 medium under a temperature of 28 ± 1 °C, light intensity of 2000–2500 lx, and light–dark cycle of 12 h on and 12 h off.

### 2.3. Culture Medium and Experimental System

Nutrient broth medium (Huankai, Guangzhou, China) was used as the culture medium and consisted of 10 g of peptone, 3 g of beef extract, 5 g of sodium chloride, 1 L of distilled water, and 20 g of nutrient agar. For the quantification of *B. cereus*, the selective medium Mannitol Yolk Polymyxin Agar Plate (MYP-Huankai, Guangzhou, China) was used, which consisted of 10 g of peptone, 1 g of beef extract, 10 g of D-mannitol, 10 g of sodium chloride, 0.026 g of phenol red, 50 mL of 50% egg yolk solution, 100,000 IU of polymyxin B, 15 g of agar, and 950 mL of distilled water.

The sulfate-type saline–alkali water system consisted of 2.365 g of Na_2_SO_4_ (Aladdin, Shanghai, China), 0.537 g of NaCl (Aladdin, Shanghai, China), 0.562 g of MgCl_2_ (Aladdin, Shanghai, China), 0.318 g of NaHCO_3_ (Aladdin, Shanghai, China), 1.113 g of CaCl_2_ (Aladdin, Shanghai, China), 0.029 g of KCl (Aladdin, Shanghai, China), 5 g of glucose (Saitek, Haikou, China), 40 mL of feed leachate, and 1 L of distilled water (Watsons, Guangzhou, China). The feed leachate was prepared as follows: grinding 100 g of commercially available shrimp feed into powder, adding 1 L of distilled water, and letting it stand in a 30 °C constant-temperature incubator for 24 h. Then, the supernatant was collected and added to the saline–alkali water system at a concentration of 40 mL/L. All the culture media used in the experiment were sterilized at 121 °C for 20 min.

For the bacterial–algal co-culture system, the initial algal concentration was set to 2.00 × 10^6^ cell/mL, and the initial bacterial concentration was 1.86 × 10^7^ CFU/mL. The system was divided into two groups: control and PT1 groups, with four replicates in each. After adding the *M. aeruginosa* algal solution, the experimental group was supplemented with an appropriate amount of bacterial solution, whereas the control group received an equal amount of sterile water. The total volume of the algal–bacterial co-culture system was 100 mL. The BG11 medium (Haibo, Qingdao, China) consisted of 1.5 g of NaNO_3_, 40 mg of K_2_HPO_4_, 75 mg of MgSO_4_·7H_2_O, 36 mg of CaCl_2_·2H_2_O, 6 mg of citric acid, 6 mg of ferric ammonium citrate, 1 mg of Na_2_EDTA, 40 mg of Na_2_CO_3_, 1 mL of A5 solution, and 999 mL of distilled water. The A5 solution consisted of 286 mg of HBO_3_, 186 mg of MnCl_2_·H_2_O, 22 mg of ZnSO_4_·7H_2_O, 39 mg of Na_2_MoO_4_·2H_2_O, 8 mg of CuSO_4_·5H_2_O, 5 mg of Co(NO_3_)_2_·6H_2_O, and 100 mL of seawater with a salinity of 5, and was used to cultivate *M. aeruginosa* [40].

### 2.4. Experimental Methods

#### 2.4.1. Quantitative Determination of *Microcystis aeruginosa* and *Bacillus cereus*

The algal–bacterial saline–alkaline solution was cultured in a light incubator (temperature 28 °C, light intensity 2000–2500 lx, light–dark cycle 12 h:12 h) for a test period of 4 days. Samples were collected every 24 h. *M. aeruginosa* samples were fixed with 5% formaldehyde solution, and the number of algal cells was counted using a hemocytometer under an optical microscope. Following the “Food Microbiological Examination—Examination of *Bacillus cereus*” standards (GB4789.14-2014 [41]), *B. cereus* counts were determined by plating on mannitol–vitelline–polymyxin agar. The changes in the appearance and color of the algal bottles were observed daily [40].

#### 2.4.2. Calculation of Algae Dissolution Rate

The algicidal rates *R* (%) for each experimental group were calculated according to the following formula [14]:

$$R(\%)=\frac{C_{0}-C_{t}}{C_{0}}\times 100$$

where *C*_0_ represents the initial concentration of *M. aeruginosa* cells in the co-culture system (cell/mL) and *C_t_* represents the concentration of *M. aeruginosa* cells on the *t*-th day of algal–bacterial co-culturing (cell/mL).

#### 2.4.3. Analysis of Differential Metabolites During Algae-Lysing

During the co-culturing of bacteria and algae, 10 mL samples were collected from both the control and experimental groups on days 0, 2, and 4 and grouped accordingly. The control group samples collected on days 0, 2, and 4 were denoted as C0, C2, and C4, respectively, whereas the experimental group samples collected on the same days were denoted as PT1_0, PT1_2, and PT1_4, respectively. These samples were then sent to the Shanghai Biotree Biomedical Technology limited company (located in Shanghai, China) for metabolomic detection and analysis.

### 2.5. Data Processing and Statistical Analysis

#### 2.5.1. Untargeted Metabolomics Data Preprocessing and Annotation

Raw LC–MS files were converted to mzXML format via ProteoWizard and subsequently processed using an R-based XCMS workflow for peak detection, retention-time alignment, and feature integration. To monitor analytical stability, pooled quality control (QC) samples—created by combining equal aliquots of all biological samples—were injected at regular intervals throughout the analytical sequence. To ensure data robustness, a stringent filtering criterion was applied: features detected in less than 80% of either the QC samples or the experimental groups were discarded. Missing values resulting from alignment gaps or low-abundance signals were addressed using k-nearest neighbors (KNN) imputation. Metabolite annotation was conducted by matching MS1 accurate mass and MS/MS fragmentation spectra against the in-house BiotreeDB (v2.1) and public databases (e.g., mzCloud, HMDB), applying a conservative spectral matching algorithm score cutoff of >0.3 to ensure annotation reliability.

#### 2.5.2. Normalization and Multivariate Analysis

Prior to statistical modeling, feature intensities were subjected to total peak area normalization to minimize non-biological technical variations. The normalized data (n = 8643 features; 4 biological replicates per group per time point) were further subjected to Log_2_(x + 1) transformation to stabilize variance, followed by Pareto scaling. Unsupervised Principal Component Analysis (PCA) was initially deployed to visualize global sample clustering and evaluate QC tightness. For supervised modeling, Orthogonal Partial Least Squares Discriminant Analysis (OPLS-DA) was utilized to maximize group separation, with model robustness and overfitting risks rigorously evaluated through 200-iteration permutation testing.

#### 2.5.3. Time-Series Differential Screening and Pattern Recognition

To identify dynamic metabolic alterations, intra-group temporal comparisons were performed. Differential features were evaluated using two-sided Welch’s *t*-tests, as it accounts for unequal variances commonly observed in metabolomics data. To control for multiple testing, *p*-values were adjusted using the Benjamini–Hochberg False Discovery Rate (BH-FDR). Volcano plots were generated utilizing thresholds of absolute log_2_FC > 1.0 and FDR < 0.05.

Specifically, we designed a rigorous dual-condition filtering strategy to isolate “PT1-induced accumulated features”. A feature was strictly defined as such only if it met both of the following criteria: (i) significant upregulation at day 2 relative to day 0 in the PT1 group (FDR < 0.05, log_2_FC > 0); (ii) persistent significant upregulation at day 4 relative to day 0 in the PT1 group (FDR < 0.05, log_2_FC > 0). Crucially, to eliminate baseline physiological drift or environmental confounders associated with prolonged cultivation, any features displaying a parallel temporal accumulation trend in the control group (both C2 vs. C0 and C4 vs. C0 exhibiting FDR < 0.05 and log_2_FC > 0) were systematically excluded from the candidate pool.

Based on this joint filtering strategy, a refined set of 298 PT1-specifically accumulated metabolic features was identified, of which 107 possessed high-confidence MS1/MS2 annotations (Metabolite identities were reported following MSI guidelines: level 1 required confirmation with authentic standards (RT and MS/MS match), whereas level 2 annotations were based on MS/MS library matching; MS1-only matches were reported as level 3). To highlight the most biologically relevant candidates, these 107 metabolites were ranked by their average magnitude of upregulation across the cultivation period (0–2 d and 0–4 d). The top 30 candidates with robust annotation evidence and pronounced differential amplitudes were selected (Table 1). To visualize their temporal accumulation trajectories, the mean group abundances of these top 30 metabolites across all time points were standardized using row Z-scores and subjected to hierarchical clustering.

#### 2.5.4. General Statistical Analysis

Non-metabolomics experimental data were analyzed and visualized using GraphPad Prism (v10.1.2) and are expressed as mean ± standard deviation (SD). Data normality and homoscedasticity were assessed prior to significance testing. Group comparisons were executed via one-way Analysis of Variance (ANOVA) with appropriate post hoc tests for parametric data, or non-parametric alternatives (e.g., Kruskal–Wallis test) when assumptions were violated. Bivariate correlations were evaluated using Pearson’s or Spearman’s correlation coefficients, contingent upon data distribution. Statistical significance was designated at *p* < 0.05, with *p* < 0.01 considered highly significant.

## 3. Results

### 3.1. Changes in the Number of Microcystis Aeruginosa Cells

**Figure 1:**
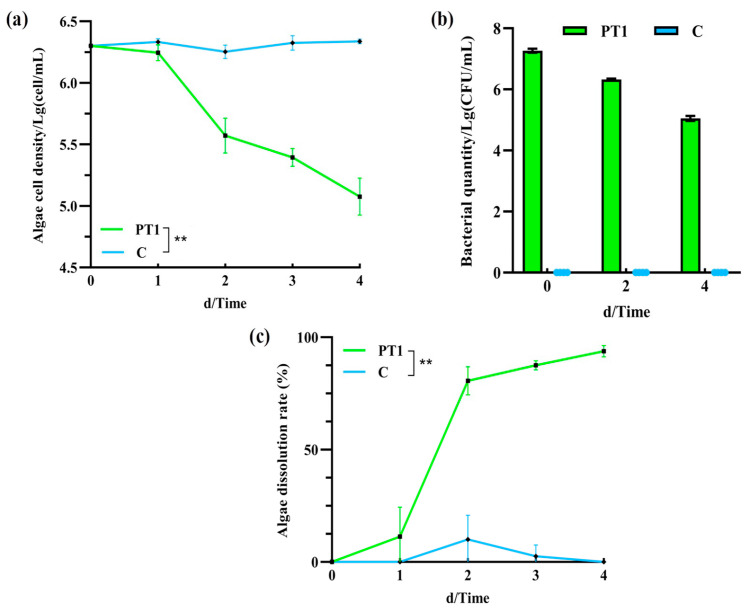
Algal cell count (**a**), *Bacillus cereus* population (**b**), and algicidal rates (**c**). Note: “**” means *p* < 0.01.

The number of *M. aeruginosa* cells in the inoculated group (PT1) was significantly lower than that in the control group (C) (*p* < 0.05). On day 2, it decreased from 2 × 10^6^ to 10^5^ cell/mL, and by day 4, it had decreased to 1.25 ± 0.5 × 10^5^ cell/mL (Figure 1a), and the difference in cell density between 2–4 days was significant (*p* < 0.05). Meanwhile, the algal biomass in the control group (C) remained stable throughout the experiment, at approximately 2.18 ± 0.95 × 10^6^ cell/mL.

### 3.2. Variation in the Bacillus cereus Population

The initial concentration of *B. cereus* in the PT1 group was 1.86 ± 0.27 × 10^7^ CFU/mL, which exhibited a decreasing trend from day 0 to day 4, reaching 5.05 ± 0.21 × 10^5^ CFU/mL on day 4 (Figure 1b). No *B. cereus* was detected in the control group.

### 3.3. Algicidal Rates

In the algal–bacterial co-culture system, the algicidal rate of the inoculated group (PT1) was 80.63% on day 2 and reached 93.75 ± 2.5% on day 4 (Figure 1c).

### 3.4. Color and Appearance Changes of Algal Bottles

**Figure 2:**
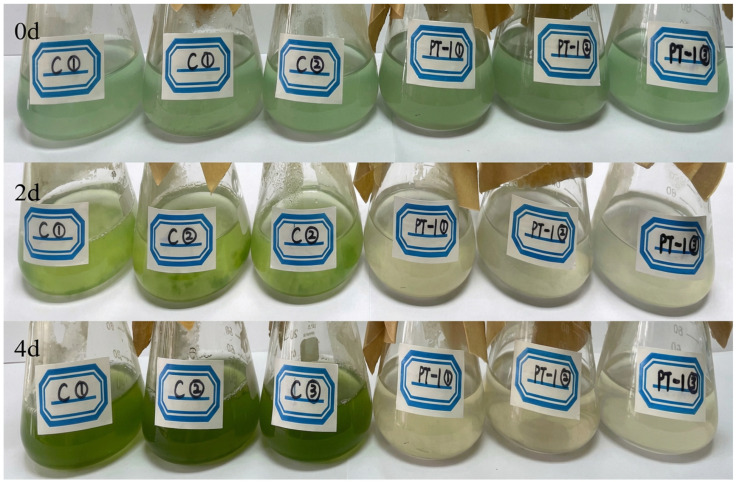
Changes in color and appearance of algal bottles.

The change in the color of the algal–bacterial co-culture system is shown in Figure 2. On day 0, both the control (C) and inoculated (PT1) groups were light green in color. As the algal–bacterial co-culture progressed, the color of the control group (C) gradually deepened from light to dark green by day 4, whereas that of the inoculated group (PT1) gradually faded from light green to yellowish-white on day 4, with yellowish-white flocculent material appearing at the bottom.

### 3.5. Metabolomics Analysis

#### 3.5.1. PCA

**Figure 3:**
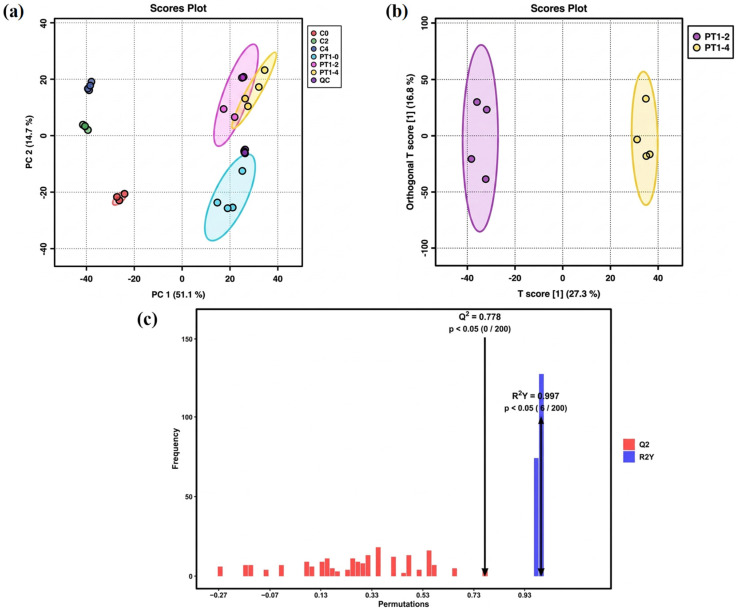
PCA score plot (**a**), OPLS-DA model scatter plot (**b**), and permutation test bar chart of the OPLS-DA model (**c**).

The metabolic profile is susceptible to external factors and changes rapidly. To obtain highly accurate metabolic profile data, QC is necessary. After Pareto-scaling processing, the peaks extracted from experimental and QC samples were subjected to total sample PCA. The smaller the distance between QC samples and the tighter their clustering, the better the experimental reproducibility and the smaller the sample differences. As shown in Figure 3a, the tight clustering of QC samples in the total ion mode indicates that the data detection method in this experiment was stable, and the data quality was high. The significant differences between the three control groups, C0, C2, and C4, indicate that the data quality of the control group measurement was reliable.

The PT1-2 and PT1-4 groups in the inoculated group showed partial overlap on axis PC1. Statistical analysis of unsupervised models could not visually indicate the differences between the two groups. Therefore, multivariate statistical analysis of the metabolites in the PT1-2 and PT1-4 algae-lysing systems was achieved via OPLS-DA in the full-ion mode for supervised pattern recognition, which effectively eliminated irrelevant influences and screened for differential metabolites. In the unsupervised PCA model, the separation trend was not evident, but the supervised OPLS-DA model achieved better separation results (Figure 3b). After 200 permutation tests, the model exhibited strong fit (R^2^Y = 0.997) and acceptable predictive ability (Q^2^ = 0.778). The permuted models consistently showed lower performance than the original model, and model reliability was examined by permutation testing (n = 200; Figure 3c). In the permutation test, none of the permuted models exceeded the original Q^2^ (0/200), corresponding to an empirical *p* value of *p* < 0.005, while 6 out of 200 permuted models reached or exceeded the original R^2^Y, giving an empirical *p* value of *p* = 0.030. Together with the QC clustering in PCA, these results support stable measurement performance and justify using the OPLS-DA output as a variable-screening aid for distinguishing PT1-2 from PT1-4.

#### 3.5.2. Metabolite Classification Statistics and Annotation

To analyze the biological functions of each metabolite, functional and taxonomic annotations were performed, and a total of 394 metabolites were annotated in the HMDB.

**Figure 4:**
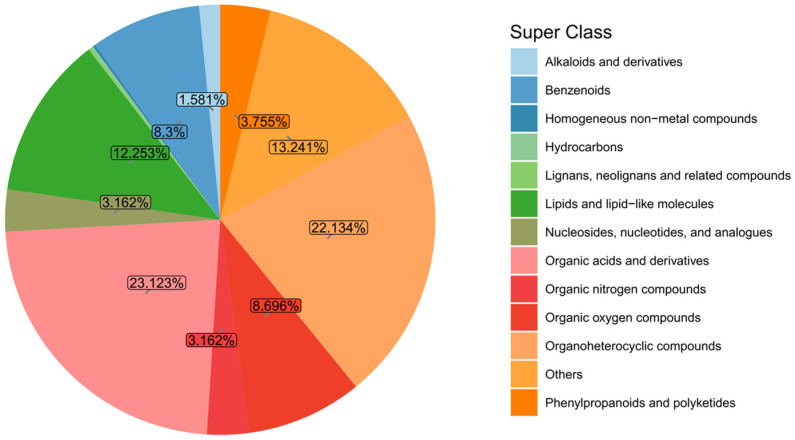
Metabolite classification and proportion pie chart. Note: Homogeneous non-metallic compounds, hydrocarbons and lignans, neo-lignans and related compounds account for 0.593%.

The identified metabolites were classified and analyzed based on their chemical classification attribution information. Figure 4 shows the proportions of the primary classifications of various metabolites in the algae-lysing system, reflecting the classification of chemical substances detected in the system. Among them, organic acids and derivatives and organoheterocyclic compounds accounted for relatively high proportions, at 23.12% and 22.13% respectively; lipids and lipid-like molecules, benzeneoids, and organic oxygen compounds followed closely, accounting for 12.25%, 8.3%, and 8.69%, respectively.

**Figure 5:**
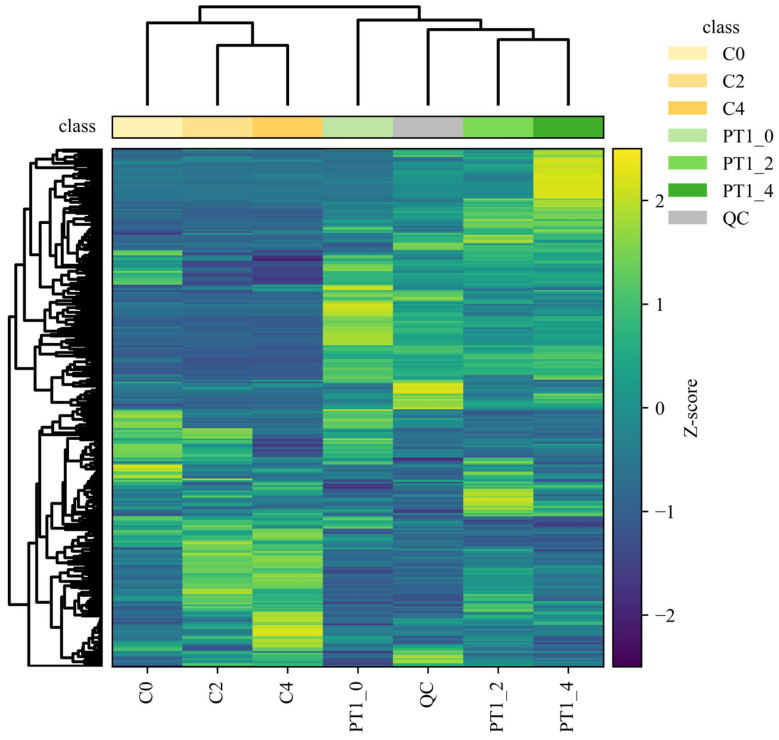
Hierarchical clustering heatmap of the PT1 and control groups.

Differences in metabolite accumulation patterns were further summarized using a cluster heatmap based on unsupervised hierarchical clustering (Pearson correlation as the distance metric; average linkage). In Figure 5, biological replicates grouped tightly, consistent with high within-group concordance (pairwise Pearson r > 0.95) and low technical variability (CV < 12%), while pooled QC injections clustered on a distinct branch, supporting stable analytical performance across the run.

To move beyond a purely descriptive interpretation, we quantified the separation of sample classes implied by the dendrogram by comparing within-group versus between-group similarity distributions. Specifically, for each group we calculated the distribution of pairwise Pearson correlations among replicates and contrasted it with correlations computed across groups; the observed within-group correlations were consistently higher than between-group correlations, confirming that the clustering pattern reflected structured group differences rather than stochastic variation. In line with this, the control samples (C0, C2 and C4) formed a coherent clade, whereas PT1-treated samples separated from controls at the global-profile level. Within the PT1-treated branch, PT1_2 and PT1_4 clustered more closely, indicating greater similarity in their late-stage metabolic profiles. Moreover, the heatmap revealed multiple feature blocks with coordinated shifts in relative abundance between PT1-treated and control samples; these shifts were consistent with a temporally staged response, as reflected by the distinct clustering of time points within each treatment.

#### 3.5.3. Screening of Differential Metabolites

**Figure 6:**
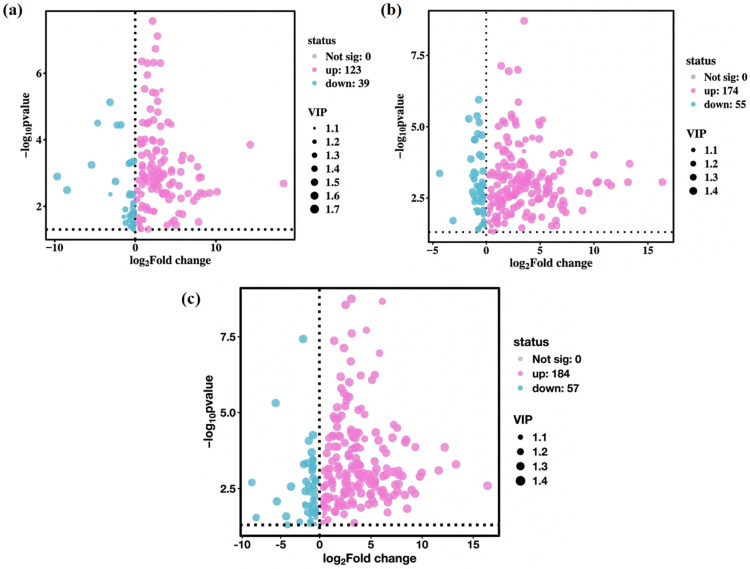
Volcanic plots of differential metabolites in the PT1 and control groups at 0, 2, and 4 d (**a**–**c**).

Owing to the high dimensionality and correlation among variables in metabolite data, multivariate statistical analysis methods such as PCA and PLS-DA are commonly used in metabolomic data analyses. These methods perform dimensionality reduction and regression analysis on multidimensional data while retaining the original information to the greatest extent, followed by facilitating the screening and subsequent analysis of differential metabolites. As shown in Figure 6a–c, 298 differential metabolites were screened in this study, including 162 between PT1_0 and C0, with 123 upregulated and 39 downregulated; 229 between PT1_2 and C2, with 174 upregulated and 55 downregulated; and 241 between PT1_4 and C4, with 184 upregulated and 57 downregulated.

**Figure 7:**
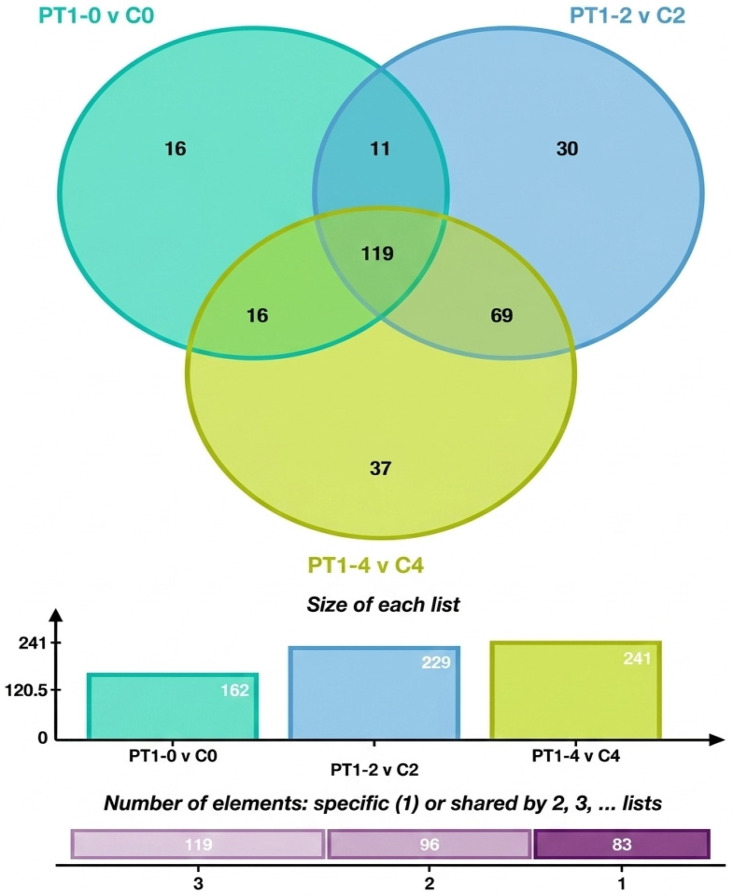
Venn diagram of total and unique metabolite counts in the PT1 and control groups at 0, 2, and 4 d.

To analyze the characteristic metabolites in the algae-lysing system that may possess algae-lysing properties, we explored the trends of differential metabolites based on the comparison between the experimental and control groups across different days. The differential metabolites from multiple comparison combinations were displayed using Venn diagrams, which visually represented the common and unique differential metabolites among different groups. Figure 7 shows 16 unique differential metabolites between PT1-0 and C0, 30 between PT1_2 and C2, and 37 between PT1_4 and C4.

To extract PT1-associated signals that accumulated over time while minimizing confounding by time-dependent drift in controls, we performed an intra-group time-series screen on the untargeted LC–MS feature table (n = 8643 features; four biological replicates per time point per group). Feature intensities were log_2_(x + 1) transformed, and PT1_2 vs. PT1_0 and PT1_4 vs. PT1_0 were tested using two-sided Welch’s *t*-test with BH-FDR correction applied per comparison. A feature was defined as a PT1-induced cumulative feature when it increased at both 2 d and 4 d relative to 0 d (FDR < 0.05 and log_2_FC > 0 in both contrasts). To further exclude generic time-driven increases unrelated to PT1, we removed features that met the same “increase at both time points” criteria in the control trajectory (C2 vs. C0 and C4 vs. C0; FDR < 0.05 and log_2_FC > 0 for both). This procedure yielded 298 PT1-induced cumulative features, of which 107 had MS1/MS2 annotation information.

Because the above definition is based on within-group time contrasts followed by subtraction of control trends, we additionally tested whether PT1 altered temporal trajectories using a formal treatment × time interaction model on log_2_(x + 1)-transformed intensities (time treated as a categorical factor). Interaction effects were quantified as difference-in-differences contrasts: (PT1_2 − PT1_0) − (C2 − C0) and (PT1_4 − PT1_0) − (C4 − C0), with BH-FDR correction applied per contrast across the 107 annotated candidates. Under this framework, 89/107 candidates showed an FDR-supported interaction at 2 d (Int_FDR_2v0 < 0.05), 103/107 at 4 d (Int_FDR_4v0 < 0.05), and 89/107 were supported at both time points, indicating that the majority of annotated cumulative features followed PT1-specific temporal trajectories rather than parallel time trends shared with controls (Table S1).

To avoid overstating identities, annotations were reported using MSI confidence levels. No compound was treated as confirmed MSI level 1 in the absence of authentic standards (retention time + MS/MS match). Among the 107 annotated PT1-induced cumulative features, 40 were supported by MS/MS library matching (reported as MSI level 2) and 67 were based on MS1 exact-mass evidence only (reported as MSI level 3); no entries were upgraded to MSI level 1 without standards (Table 1 and Table S1).

**Figure 8:**
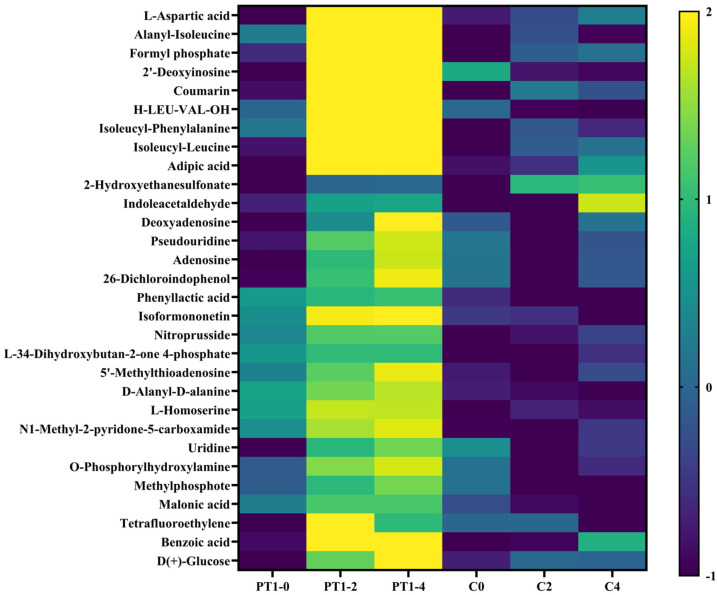
Heatmap of the Top 30 differentially expressed metabolites.

To prioritize interpretable candidates, we ranked annotated cumulative features by the mean upregulation magnitude across PT1_0→PT1_2 and PT1_0→PT1_4 and selected the top 30 (Table 1). Within these top candidates, 16/30 were MSI level 2 and 14/30 were MSI level 3. Using row Z-score normalization of group means and hierarchical clustering, these metabolites exhibited a coherent accumulation pattern in the PT1 condition between 2–4 d, whereas the control did not display the same two-time-point increase pattern, resulting in distinguishable PT1-associated signatures at the profile level (Figure 8). Consistent with the interaction analysis, 26/30 top candidates were supported by treatment × time interaction at both 2 d and 4 d (Int_FDR_2v0 < 0.05 and Int_FDR_4v0 < 0.05; Table S1).

Finally, we assessed whether any top candidates were consistent with published algicidal/anti-cyanobacterial chemistry or plausible physiological targets, while treating these links as mechanistic hypotheses rather than causal proof. For example, benzoic acid has been reported as an active small lytic molecule produced by marine algicidal bacteria that can induce algal cell rupture and lead to the leakage of contents [30]; additionally, coumarin and malonic acid have been verified in allelopathic/algicidal studies to have inhibitory effects on algal growth [42].

Some candidates were more likely to reflect “key physiological process clues during the algal lysis stage,” rather than be confirmed as algal lysis toxins themselves: for example, studies involving exogenous NO donors suggest that NO can inhibit photosynthetic electron transfer in cyanobacteria [43]; similarly, phosphonates have been reported to cause iron limitation and inhibit microalgal growth and photosynthetic function by forming complexes with iron [44]. Furthermore, the indole/indole-3-acetic acid (IAA)-related pathway is believed to be involved in algal–bacterial cross-border signal communication, providing an explanatory framework for the “signal molecule dimension” of some candidates [45].

Collectively, direct algicidal activity is documented for only a subset of candidates, whereas many others may reflect metabolic remodeling or signaling during PT1-mediated lysis; therefore, follow-up work will prioritize (1) upgrading key annotations to higher MSI confidence using standards and targeted MS/MS and (2) functional assays with purified compounds (alone and in combinations) to test whether they act as causal chemical drivers of the lytic phenotype.

#### 3.5.4. KEGG Pathway Enrichment Analysis

**Figure 9:**
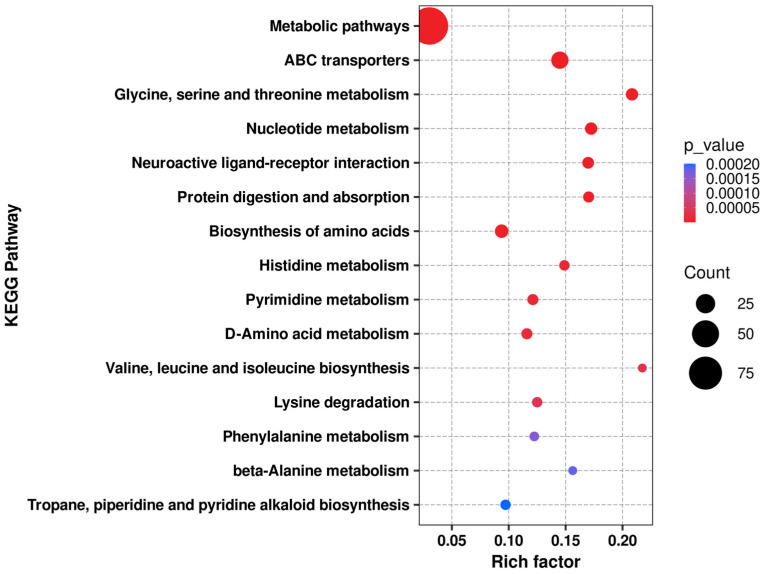
KEGG enrichment bubble chart of the PT1 and control groups.

**Figure 10:**
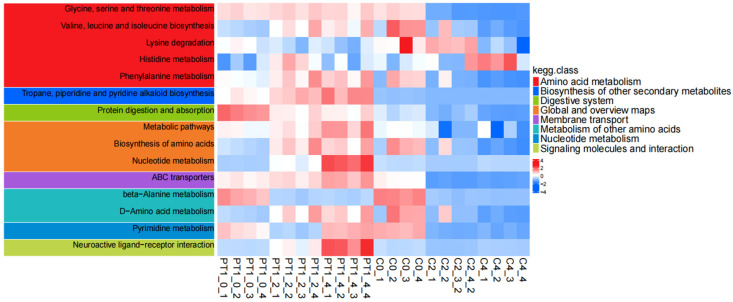
Time-series KEGG annotation heatmaps of the PT1 and control groups.

After obtaining the “PT1-induced accumulation” metabolic characteristics and screening the Top 30 candidates, we further conducted KEGG pathway enrichment analysis on the differential metabolites between the PT1 group and control group C at three time points: 0, 2, and 4 d using an over-representation analysis based on the hypergeometric distribution (Fisher’s exact test). Raw *p* values were adjusted at the pathway level using the Benjamini–Hochberg procedure to obtain q values (BH-FDR), and pathways with q < 0.05 were considered significantly enriched and visualized in bubble plots and heatmaps (Figure 9 and Figure 10).

KEGG pathway enrichment analysis revealed systematic changes in pathways related to amino acid and nucleotide metabolism, as well as transmembrane transport, during the key algae lysis stage following PT1 treatment. Furthermore, the enrichment direction at the pathway level was consistent with the “stable accumulation signal” reflected by the Top 30 candidates screened from differential metabolites regarding functional direction. It is worth noting that the Top 30 candidate compounds have a high proportion of purine metabolism (map 00230), nucleotide metabolism (map 01232), and pyrimidine metabolism (map 00240), as well as cysteine and methionine metabolism (map 00270). Overall, the Top 30 candidates did not change in isolation, but were embedded within a group of synergistic pathways centered on amino acid/nucleotide metabolism and transmembrane transport (Table S2).

According to the KEGG enrichment analysis results of PT1 and the control group (C), the differential metabolites were mainly enriched in pathways related to amino acid and nucleotide metabolism (Figure 9). Among all enriched functions, “metabolic pathways” had the highest number of differential metabolites, but its Rich factor was relatively low, suggesting that this functional pathway reflects a broader “global metabolic pathway”-level enrichment. In contrast, the glycine/serine/threonine metabolism, valine/leucine/isoleucine biosynthesis, ABC transporter, and nucleotide-related metabolic pathways exhibited higher degrees of enrichment, often with higher significance; some pathways, such as phenylalanine metabolism and tropane/piperidine/pyridine alkaloid biosynthesis, showed relatively weaker significance but still had certain enrichment signals.

The time-series KEGG pathway heatmap (0/2/4 d) further demonstrates that these enriched functions were not static but dynamically changed with treatment and time. Specifically, pathways related to amino acid metabolism, such as glycine/serine/threonine metabolism, branched-chain amino acid biosynthesis, and lysine, histidine, and phenylalanine metabolism, as well as nucleotide metabolism (including pyrimidine metabolism), exhibited systematic fluctuations in pathway-level scores at different time points, and indicated clearer overall differences between the PT1 and control groups in the later stages. Additionally, pathways related to membrane transport function (ABC transporters) and overall biosynthetic capacity (such as the overview of amino acid biosynthesis and metabolism pathways) also exhibited coordinated changes, suggesting that PT1 treatment may be accompanied by the reshaping of the core metabolic network.

In summary, compared with the control group, the differentially expressed metabolites in the PT1-inoculated group were more prone to affecting the central metabolic network, particularly focusing on amino acid metabolism, nucleotide metabolism, and transport-related functions. Furthermore, after incorporating the time dimensions of 0/2/4 d, this pathway-level difference became even more pronounced.

#### 3.5.5. Screening of Algae Lysis-Related Pathways

Based on a systematic review and the current research on algicidal bacteria, the algicidal-related functions summarized under the KEGG framework can be categorized into three types (Table 2): First, the regulatory module, primarily encompassing quorum sensing (QS) and environmental signal response (such as QS-map02024 and two-component system (TCS)-map02020), explains the common phased and threshold effects of algicidal phenotypes and their sensitive responses to external conditions. Second, the substance export and transmembrane transport module, represented by ABC transporters (map02010), supports the transport and accumulation of extracellular active factors/metabolites during the algicidal process. Notably, this enrichment is based on metabolite-to-KEGG annotation and should be interpreted as a pathway-level association, not direct proof of ABC-transporter activation or causality in metabolite export/accumulation. Finally, the third includes active small molecules and their precursor metabolic modules, often involving aromatic and indole metabolism (such as tryptophan metabolism-map00380, and degradation of aromatic compounds-map01220), metabolism of amino acids and their derivatives (such as lysine biosynthesis-map00300, and lysine degradation-map00310), as well as terpene backbone biosynthesis and isoprene precursor supply (terpenoid backbone biosynthesis-map00900). These pathways provide a reasonable metabolic background and biosynthetic sources for various reported algicidal active substances.

Based on existing pathway clues and the enrichment analysis and screening of the metabolic pathways of PT1 vs. C, the enrichment results of differential metabolites during the induced algae lysis process in the PT1 group suggest that its metabolic response was concentrated around three interconnected functional chains: First, at the regulatory level, enrichment was observed in both QS (map02024) and TCS (map02020), indicating changes in networks related to cell density-dependent regulation and environmental signal responses, which is consistent with the enhanced algae lysis effect with cultivation time. Second, at the substance output level, ABC transport (map02010) was enriched, suggesting that the differential metabolites were not only involved in intracellular metabolic rearrangement, but were also possibly related to small-molecule transmembrane transport/extracellular accumulation, thereby aligning with the common pattern of “secreted or diffusible active factors participating in algae lysis.” Third, at the level of candidate effector molecules and their precursor metabolism, tryptophan metabolism (map00380) and degradation of aromatic compounds (map01220) were enriched, indicating involvement in the transformation process of indole/aromatic derivatives. Meanwhile, lysine biosynthesis and degradation (map00300/map00310) indicates that amino acid-related metabolic fluxes are affected, whereas terpenoid backbone biosynthesis (map00900) indicates that metabolism related to isoprene precursor supply may have been involved in constructing and regulating part of the secondary metabolite background.

Comprehensively, the algae-lysing process of the inoculated group PT1 was characterized by the emergence and enhancement of the algae-lysing phenotype, accompanied by the enrichment of pathways centered around “regulation (QS/TCS)–transport (ABC)–small-molecule metabolism (indole/aromatic compounds, amino acids, and terpene precursors)” in the algae-lysing active metabolites secreted by PT1.

**Table 2:** Summary of algicidal substances and related pathways.

Algicidal Bacterium	Algicidal Compound	KEGG Pathway ID
*Shewanella* sp. Lzh-2 [46]	Hexahydropyrrolo[1,2-a]pyrazine-1,4-dione; Isatin (1H-indole-2,3-dione)	map00380—Tryptophan metabolism
*Bacillus* sp. Lzh-5 [47]	Hexahydropyrrolo[1,2-a]pyrazine-1,4-dione; 3-isopropyl-hexahydropyrrolo[1,2-a]pyrazine-1,4-dione	map01054—Non-ribosomal peptide structures
*Stenotrophomonas* sp. F6 [48]	Cyclo-(Gly-Pro); Hydroquinone	map00350—Tyrosine metabolism; map00362—Benzoate degradation
*Bacillus* sp. S51107 [49]	Indole-3-carboxaldehyde; Cyclo(Pro-Phe) (cyclic dipeptide)	map02024 (Quorum sensing)
*Streptomyces* sp. L74 [50]	A triterpenoid saponin	map00909—Sesquiterpenoid and triterpenoid biosynthesis; map00900—Terpenoid backbone biosynthesis
*Streptomyces phaeofaciens* [51]	L-Lysine	map00300—Lysine biosynthesis; map00310—Lysine degradation
*Hahella* sp. KA22 [52]	Prodigiosin	map00333—Prodigiosin biosynthesis
*Pseudomonas* sp. QJX-1 [53]	2,4-di-tert-butylphenol	map01220—Degradation of aromatic compounds
*Bacillus tequilensis* strain D8 [54]	Surfactin homologues (C13/C14/C15)	map01054—Nonribosomal peptide structures
*Pseudomonas aeruginosa* [55]	Rhamnolipid biosurfactants	map00541—Biosynthesis of various nucleotide sugars
*Phaeobacter gallaeciensis* [56]	Roseobacticides	map02024—Quorum sensing

## 4. Discussion

### 4.1. Saline–Alkali Tolerance and High Algae-Lysing Potential of Strain PT1

Owing to its high pH, high salinity, and complex ionic strength, saline–alkaline water often exerts strong osmotic pressure on microorganisms, limiting the colonization and activity of conventional freshwater algicidal bacteria (such as *Pseudomonas* sp. and *Aeromonas* sp.). In this study, the indigenous strain PT1 isolated from a saline–alkaline habitat exhibited excellent adaptability in a co-culture system, achieving a high *M. aeruginosa* removal rate of 93.75% by day 4. Supporting the feasibility of an “indigenous strain” strategy for cyanobacterial control under saline–alkaline conditions.

Notably, while direct cross-study comparisons should be made cautiously due to differences in experimental settings (e.g., inoculation ratio, light regime, algal density, and water matrix), the removal efficiency observed for PT1 is at the higher end of values reported for several algicidal bacteria evaluated under laboratory conditions. For example, an *Enterobacter* strain (H6) showed ~70% removal after 7 days at its optimal dosage, and 70–80% removal after 7 days across pH 5–11 [57]. A *Chitinimonas* strain (G1) achieved a maximum algae-lysis rate of 67.64% under a 15% dosing ratio and 68.21% in darkness [58]. In another study, immobilized *Bacillus* sp. HL reached an algicidal rate of 77.67% ± 1.14% by day 7 [59]. Together, these published benchmarks provide quantitative context suggesting that PT1 displays strong lytic potential in the saline–alkaline co-culture setting. This discovery is highly consistent with recent research on saline–alkaline-tolerant algicidal bacteria and validating the effectiveness of the “indigenous microbial management” strategy. For example, Jiang et al. [40] isolated *B. cereus* TC-1, which exhibited strong growth stability and algicidal activity in sulfate-type saline–alkaline water. Additionally, Hu et al. [14] studied the algicidal bacterium CZBC1 and found that it had a longer algicidal effect on *M. aeruginosa* in chloride-type saline–alkaline water, which may have been related to the strain’s isolation and optimal growth environment. The *Bacillus* genus can balance high saline–alkaline osmotic pressure by regulating intracellular solutes (such as betaine and proline), thereby maintaining algicidal substance synthesis and secretion [60]. The stable performance of PT1 in this study further confirms that the *Bacillus* genus could be a dominant germplasm resource for developing cyanobacteria control agents in saline–alkaline water bodies.

### 4.2. Analysis of Potentially Active Substances Based on Metabolomics

Using non-targeted metabolomics combined with strict multivariate statistical analysis (OPLS-DA), a group of characteristic metabolites closely related to the algicidal phenotype in existing studies were identified. Based on this, we hypothesize that these metabolic candidates form a multi-target attack network, consisting of the following parts:

Organic Acid Stress: We detected significant accumulation of benzoic and malonic acid. Early research by Chai et al. [61] indicated that benzoic acid has a significant inhibitory effect on cyanobacteria growth, and Liu et al. [62] further revealed that its mechanism involves the disruption of cell membrane permeability and inhibition of photosystem II (PSII) activity. The latest research by Tao et al. [63] demonstrated that malonic acid as a key algicidal factor, confirming that it can induce severe oxidative stress (ROS) and lipid peroxidation in algal cells.

Signaling Interference: The identification of IAA and its derivatives holds significant importance. Although IAA is commonly regarded as an auxin in plants, it serves as a cross-border signaling molecule in the interaction between algae and bacteria. Duca et al. [64] reported that IAA secreted by bacteria can disrupt the cell cycle of microalgae at high concentrations, inducing programmed cell death (PCD). This suggests that PT1 may utilize hormone analogues to interfere with the physiological homeostasis of algae.

Potential algicidal peptide precursor: Zhang et al. [65] reported that *Bacillus* produces large amounts of lipopeptide antibiotics such as Surfactin and Iturin, which are assembled from amino acid modules and exhibit broad-spectrum activity in biofilm disruption. To test whether benzoic acid, malonic acid and IAA-related compounds act as causal chemical drivers, future work will prioritize (1) standard-based targeted confirmation (retention time and MS/MS) and (2) controlled bioassays using purified compounds across concentration gradients to obtain dose–response relationships, ideally under matched saline–alkaline matrix conditions.

### 4.3. Metabolic Remodeling Induced by Strain PT1 and Its Potential Algicidal Effect

In this study, the pronounced algal-lysing phenotype observed in the PT1-treated group between days 2 and 4 was temporally concordant with a system-wide remodeling of cellular metabolism. KEGG enrichment analysis indicated that the perturbed signals were not randomly distributed; instead, they clustered in pathways related to environmental sensing, transmembrane transport and small-molecule biosynthesis. Together, these patterns support a testable working model in which PT1 may participate in algal lysis through a “signal sensing–material transport–effector molecule production/release” axis. Importantly, pathway enrichment derived from untargeted metabolomics should not be taken as evidence of transcriptional or functional activation of the corresponding modules, and targeted gene-expression readouts and/or functional perturbation will be required to evaluate causality.

First, the co-enrichment of QS (map02024), TCS (map02020), and ABC transporter (map00380) suggests a coordinated response and enhanced exocytosis ability of algicidal bacteria under environmental signals. Previous studies have demonstrated that the algicidal activity of bacteria is often gated by cell-density-dependent QS systems [66], such as that of *Bacillus* sp., which is primarily regulated by the NprR–NprX-type QS system [49]. In this study, the simultaneous upregulation of signal transduction and transport systems may reflect that the PT1 strain initiates a similar communication mechanism during the algicidal process to coordinate effector molecule synthesis and release. At present, however, we lack direct evidence from transcriptomics, qPCR, or inhibitory/mutational assays to substantiate a causal role of these modules.

Second, the significant enrichment of amino acids and secondary metabolic pathways provides chemical indicators for the categories of potential algicidal active substances. The activities of tryptophan metabolism (map00380) and aromatic compound degradation (map01220) suggest the possibility of indole or phenolic derivative production. Previously, in algicidal bacteria such as *Shewanella* [46] and *Stenotrophomonas* [48], it was confirmed that aromatic metabolites such as isatin (2,3-dihydroindolinedione) and hydroquinone [67] could cause severe damage to the cyanobacteria cell structure. In addition, alterations to the lysine synthesis/degradation (map00300/map00310) pathway are noteworthy, as extracellular L-lysine is a key factor in the lysis of cyanobacterial cell walls by *Streptomyces* [51]. Meanwhile, triterpene saponin structures have been identified in freshwater algicidal *Streptomyces* bacteria, and an algicidal effect on *Microcystis* has been observed [50], indicating that the terpene skeleton can carry algicidal active molecules. The enrichment of terpene skeleton biosynthesis (map00900) in this study may suggest that the addition of PT1 could bisoprenoid precursor supply and/or associated metabolic branches may be re-directed, potentially supporting the formation of hydrophobic small molecules or lipid-like effectors. Notably, these inferences are primarily derived from pathway-level trends and do not, by themselves, resolve the chemical identity or mode of action of specific algicidal agents.

In summary, based on the bactericidal effect and metabolic characteristics of strain PT1, it is speculated that its algal killing effect is driven by the signal transduction system (QS) and transport complex system (TCS), enhanced by promoting substance exchange through the ABC transport system, and generated by potential bactericidal factors generated by the reshaped aromatic, nitrogen-containing, and terpenoid metabolic networks. Although non targeted metabolomics describes the bactericidal characteristics of PT1, candidate substances identified through non targeted metabolomics, such as benzoic acid and malonic acid, need to be further validated through pure compound supplementation. In addition, the impact of high ion strength in saline–alkali environments on the stability and toxicity of these active substances is also worth evaluating. Future research will focus on isolating active ingredients, conducting toxicological validation, and genetic modification to improve the applicability of the strain in complex saltwater environments.

In summary, the algicidal effect of PT1 is driven by the QS and TCS, enhanced by the ABC transport system for material exchange, and generated by potential algicidal effect factors utilizing the reshaped aromatic, nitrogen-containing, and terpene metabolic networks. Although non-targeted metabolomics characterized the algicidal profile of PT1, the candidates identified via non-targeted metabolomics—such as benzoic and malonic acids—require further validation through pure compound supplementation. Furthermore, the potential impact of high ionic strength in saline–alkali environments on the stability and toxicity of these active substances warrants assessment. Future investigations will focus on the isolation of active agents, toxicological verification, and genetic modification to enhance the strain’s applicability in complex saline water bodies.

## 5. Conclusions

Algicidal bacteria offer a promising biological control strategy for preventing harmful algal blooms, but their effect mechanisms in saline–alkaline environments require further study. The aim of this study was to explore the algae-lysing effects and metabolic mechanisms of the indigenous *Bacillus* strain PT1 isolated from saline–alkaline environments on *Microcystis aeruginosa*. The algae-lysing performance of PT1 was evaluated via co-culture experiments, and non-targeted metabolomics technology, combined with differential metabolite screening and KEGG pathway enrichment analysis, was utilized to elucidate the key metabolic characteristics and regulatory network induced by strain PT1. The strain exhibited significant and stable algae-lysing effects, with the number of algal cells decreasing from an initial 2 × 10^6^ to 1.25 ± 0.5 × 10^5^ cell/mL, and the algicidal rate reaching 93.75 ± 2.5% on day 4. The color of the algal solution faded from green and was accompanied by sedimentation, indicating damage to the algal cell structure. The culturable bacterial biomass in group PT1 decreased during the algae-lysing process, indicating that the process mainly relied on the accumulation of metabolites, rather than bacterial proliferation. Non-targeted metabolomics co-screening identified 298 PT1-induced cumulative metabolic characteristics, defined as LC–MS features that increased in PT1 at both day 2 and day 4 relative to day 0 (log_2_FC > 0, BH-FDR < 0.05 for PT1_2 vs. PT1_0 and PT1_4 vs. PT1_0) while not showing the same sustained increase pattern in controls. Among which the top 30 metabolites included organic acids/aromatic compounds such as benzoic acid, coumarin, and malonic acid, as well as signaling-related molecules, such as indole-3-aldehyde and sodium nitroprusside, indicating the synergistic effects of membrane permeability stress, redox perturbation, and photosynthetic inhibition. KEGG pathway enrichment analysis demonstrated that the differential metabolites were significantly concentrated in eight reported algae-lysing-related pathways, including QS, TCS, ABC transporters, and tryptophan metabolism. The algae-lysing active metabolites secreted by PT1 exhibited pathway enrichment characteristics centered around “regulation (QS/TCS)–transport (ABC)–small molecule metabolism (indole/aromatic compounds, amino acids, and terpene precursors).” The above conclusion is based on existing research and speculation of the algal dissolution model at the metabolic level, lacking functional validation of candidate substances for algal dissolution, This study provides a theoretical basis for further elucidating the molecular characteristics of algae solubilizing activity under saline–alkali conditions. Therefore, future work will focus on isolating specific algicidal effectors and verifying their biosafety, followed by genetic modification to enhance the adaptability and efficacy of the strain in dynamic saline-alkali ecosystems.

## Figures and Tables

**Table 1:** Top 30 differential metabolites.

Feature ID	Metabolite Name	Feature ID	Metabolite Name
16	Adenosine	2130	2,6-Dichloroindophenol
35	2-Hydroxyethanesulfonate	312	L-Homoserine
6	Benzoic acid	4746	L-Aspartic acid
70	Phenyllactic acid	212	Pseudouridine
17	Deoxyadenosine	5178	Formyl phosphate
5252	Tetrafluoroethylene	4779	Adipic acid
67	Isoleucyl-Leucine	4780	N1-Methyl-2-pyridone-5-carboxamide
118	Isoleucyl-Phenylalanine	106	Uridine
4632	Coumarin	165	D-Alanyl-D-alanine
5157	Methylphosphote	4785	2′-Deoxyinosine
92	H-LEU-VAL-OH	2864	L-3,4-Dihydroxybutan-2-one 4-phosphate
7796	Nitroprusside	5224	O-Phosphorylhydroxylamine
76	Indoleacetaldehyde	26	Malonic acid
542	D(+)-Glucose	48	Alanyl-Isoleucine

## Data Availability

The original contributions presented in the study are included in the article/Supplementary Materials, further inquiries can be directed to the corresponding author.

## Supplementary Materials

The following supporting information can be downloaded at: https://www.mdpi.com/article/10.3390/microorganisms14030647/s1, Table S1. Statistical Table of Dual-factor Interaction Differences in Metabolites; Table S2. Top 30 Metabolite Enrichment Pathways Table.

## Abbreviations

LC–MS: Liquid Chromatography–Mass Spectrometry; log2FC: log2 Fold Change; BH-FDR: Benjamini–Hochberg False Discovery Rate; KEGG: Kyoto Encyclopedia of Genes and Genomes; QS: Quorum Sensing; TCS: Two-Component System.
