# Supplementary material for: Algicidal Characteristics of Bacillus cereus Strain PT1 Against Microcystis aeruginosa in Sulfate-Type Saline–Alkaline Environments
Source: Microorganisms. 2026 Mar 13;14(3):647. doi: 10.3390/microorganisms14030647 (PMC13028743; doi:10.3390/microorganisms14030647)
Supplement: Supplementary file 1 [file microorganisms-14-00647-s001.zip › microorganisms-4183896-supplementary.pdf]

Table S1. Statistical Table of Dual-factor Interaction Differences in Metabolites

Feature_ID	Best_Name	MS2_name	MS1_name	MSI_level	In_Top 30
6	Benzoic acid	Benzoic acid		2	TRUE
16	Adenosine	Adenosine		2	TRUE
17	Deoxyadenosine	Deoxyadenosine		2	TRUE
24	5'-Methylthioadenosine	5'-Methylthioadenosine		2	TRUE
26	Malonic acid	Malonic acid		2	TRUE
35	2-Hydroxyethanesulfonate	2-Hydroxyethanesulfonate		2	TRUE
48	Alanyl-Isoleucine	Alanyl-Isoleucine		2	TRUE
67	Isoleucyl-Leucine	Isoleucyl-Leucine		2	TRUE
70	Phenyllactic acid	Phenyllactic acid		2	TRUE
76	Indoleacetaldehyde	Indoleacetaldehyde		2	TRUE
92	H-LEU-VAL-OH	H-LEU-VAL-OH		2	TRUE
106	Uridine	Uridine		2	TRUE
118	Isoleucyl-Phenylalanine	Isoleucyl-Phenylalanine		2	TRUE
165	D-Alanyl-D-alanine	D-Alanyl-D-alanine		2	TRUE
212	Pseudouridine	Pseudouridine		2	TRUE
312	L-Homoserine	L-Homoserine		2	TRUE
542	D(+)-Glucose		D(+)-Glucose	3	TRUE
2130	2,6-Dichloroindophenol		2,6-Dichloroindophenol	3	TRUE
2864	L-3,4-Dihydroxybutan-2-one 4-phosphate		L-3,4-Dihydroxybutan-2-one 4-phosphate	3	TRUE
4629	Isoformononetin		Isoformononetin	3	TRUE
4632	Coumarin		Coumarin	3	TRUE
4746	L-Aspartic acid		L-Aspartic acid	3	TRUE
4779	Adipic acid		Adipic acid	3	TRUE
4780	N1-Methyl-2-pyridone-5-carboxamide		N1-Methyl-2-pyridone-5-carboxamide	3	TRUE
4785	2'-Deoxyinosine		2'-Deoxyinosine	3	TRUE
5157	Methylphosphote		Methylphosphote	3	TRUE
5178	Formyl phosphate		Formyl	3	TRUE

			phosphate		
5224	O-Phosphorylhydroxylamine		O-Phosphorylhydroxylamine	3	TRUE
5252	Tetrafluoroethylene		Tetrafluoroethylene	3	TRUE
7796	Nitroprusside		Nitroprusside	3	TRUE
20	Glycyl-L-Leucine	Glycyl-L-Leucine		2	
31	Phenylalanylphenylalanine	Phenylalanylphenylalanine		2	
77	N6-Isopentenyladenosine	N6-Isopentenyladenosine		2	
86	Threoninyl-Leucine	Threoninyl-Leucine		2	
96	4'-Hydroxyflurbiprofen	4'-Hydroxyflurbiprofen		2	
129	Allopurinol-1-ribonucleoside	Allopurinol-1-ribonucleoside		2	
149	Lysyl-Alanine	Lysyl-Alanine		2	
178	3,7-Dimethyluric acid	3,7-Dimethyluric acid		2	
193	N-Acetylmuramate	N-Acetylmuramate		2	
200	Phenylalanyl-Tryptophan	Phenylalanyl-Tryptophan		2	
204	1-(beta-D-Ribofuranosyl)-1,4-dihydronicotinamide	1-(beta-D-Ribofuranosyl)-1,4-dihydronicotinamide		2	
206	5-Acetyl-3,4-dihydro-2H-pyrrole	5-Acetyl-3,4-dihydro-2H-pyrrole		2	
270	L-Histidine trimethylbetaine	L-Histidine trimethylbetaine		2	
278	2-Hydroxypyridine	2-Hydroxypyridine		2	
286	5-Aminopentanamide	5-Aminopentanamide		2	
291	Biotin	Biotin		2	
310	Denudatine	Denudatine		2	
314	Valyl-Arginine	Valyl-Arginine		2	
321	Moracin I	Moracin I		2	
334	L-Allothreonine	L-Allothreonine		2	
373	L-alpha-glutamyl-L-hydroxyproline	L-alpha-glutamyl-L-hydroxyproline		2	
374	Mollicellin A	Mollicellin A		2	
379	Gallic acid	Gallic acid		2	
385	1-Deoxy-D-glucitol	1-Deoxy-D-glucitol		2	
496	(R)-Reticuline		(R)-Reticuline	3	

549	Dehydrocorydaline		Dehydrocorydaline	3	
559	(S)-10-Hydroxycamptothecin		(S)-10-Hydroxy camptothecin	3	
590	Guanosine		Guanosine	3	
591	N-Methylflindersine		N-Methylflindersine	3	
594	Nepodin		Nepodin	3	
596	Dihydropinosylvin		Dihydropinosylvin	3	
974	5'-Deoxy-5'-fluoroadenosine		5'-Deoxy-5'-fluoroadenosine	3	
1224	Calcium formate		Calcium formate	3	
1534	S-(1,2-Dichlorovinyl)-L-cysteine		S-(1,2-Dichloro vinyl)-L-cysteine	3	
1604	Methyl methylthio selenide		Methyl methylthio selenide	3	
1623	O-Phospho-L-homoserine		O-Phospho-L-homoserine	3	
1638	Validamine 7-phosphate		Validamine 7-phosphate	3	
2188	Propanil		Propanil	3	
2281	Shikimate 3-phosphate		Shikimate 3-phosphate	3	
2290	[2,6-dihydroxy-3-(3,5,6,7-tetrahydroxy-4-oxo-4H-chromen-2-yl)phenyl]oxidanesulfonic acid		[2,6-dihydroxy-3-(3,5,6,7-tetrahydroxy-4-oxo-4H-chromen-2-yl)phenyl]oxidanesulfonic acid	3	
2297	N-Phosphohypotaurocyamine		N-Phosphohypotaurocyamine	3	
2328	Fenhexamid		Fenhexamid	3	
2346	Lormetazepam		Lormetazepam	3	
2357	S-Methyl-3-phospho-1-thio-D-glycerate		S-Methyl-3-phospho-1-thio-D- glycerate	3	
2447	7,8-Dihydroneopterin 2',3'-cyclic phosphate		7,8-Dihydroneopterin 2',3'-cyclic	3	

			phosphate		
2566	Molybdopterin precursor Z		Molybdopterin precursor Z	3	
3131	Pamidrote		Pamidrote	3	
3205	ADP		ADP	3	
3321	Dichlofenthion		Dichlofenthion	3	
3925	rcobarbital		rcobarbital	3	
3959	1-Deoxy-D-altro-heptulose 7-phosphate		1-Deoxy-D-altro-heptulose 7-phosphate	3	
4079	Bluensidine 6-phosphate		Bluensidine 6-phosphate		
4514	Biopterin		Biopterin		
4560	Sepiapterin		Sepiapterin		
4561	O-Phospho-L-threonine		O-Phospho-L-threonine		
4622	Isoliquiritigenin		Isoliquiritigenin		
4627	L-Isoleucine		L-Isoleucine		
4635	beta-Hydroxypyruvic acid		beta-Hydroxypyruvic acid		
4661	Biopterin		Biopterin		
4695	Biopterin		Biopterin		
4751	O-Phospho-L-threonine		O-Phospho-L-threonine		
4774	Phloretin		Phloretin		
4778	Kynurenic acid		Kynurenic acid		
4902	4-Bromophenol		4-Bromophenol		
4986	Cobaltous sulfate		Cobaltous sulfate		
5053	Radium-224				
5094	Diclofec				
5129	Ferricyanide				
5356	3,5-Dichloro-2-methylmucote				
5361	4,6-Dichloro-3-methylcatechol				
5559	Methylphosphote				
6802	Phosacetim				
6912	3-Iodo-4-hydroxyphenylpyruvate				
6969	5-Phosphonooxy-L-lysine				

6971	Bretylium				
6996	Benoxacor				
7020	2-(Formamido)-N1-(5'-phosphoribosyl)acetamidine				

Table S2. Top 30 Metabolite Enrichment Pathways Table

**Top30 Metabolite Best Name**	KEGG Pathway	KEGG Description
D(+)-Glucose		
L-Aspartic acid		
5'-Methylthioadenosine	map00270; map01100	Cysteine and methionine metabolism; Metabolic pathways
Benzoic acid	map00362; map00621; map00627; map01100; map01110; map01120; map01220	Aminobenzoate degradation; Benzoate degradation; Biosynthesis of secondary metabolites; Degradation of aromatic compounds; Dioxin degradation; Metabolic pathways; Microbial metabolism in diverse environments
Adenosine	map00230; map01100; map01232; map02010	ABC transporters; Metabolic pathways; Nucleotide metabolism; Purine metabolism
L-Homoserine	map00260; map00270; map00300; map00920; map01100; map01110; map01120; map01230	Biosynthesis of amino acids; Biosynthesis of secondary metabolites; Cysteine and methionine metabolism; Glycine, serine and threonine metabolism; Lysine biosynthesis; Metabolic pathways; Microbial metabolism in diverse environments; Sulfur metabolism
Uridine	map00240; map01100; map01232; map02010	ABC transporters; Metabolic pathways; Nucleotide metabolism; Pyrimidine metabolism
Malonic acid	map00061; map00240; map00410; map01100; map01212	Fatty acid biosynthesis; Fatty acid metabolism; Metabolic pathways; Pyrimidine metabolism; beta-Alanine metabolism

Deoxyadenosine	map00230; map01100; map01232; map02010	ABC transporters;<br>Metabolic pathways;<br>Nucleotide metabolism;<br>Purine metabolism
Indoleacetaldehyde	map00380; map01100	Metabolic pathways;<br>Tryptophan metabolism
D-Alanyl-D-alanine	map00470; map00550; map01100; map01502	Amino acid metabolism;<br>Metabolic pathways;<br>Peptidoglycan biosynthesis;<br>Vancomycin resistance
Pseudouridine	map00240; map01100	Metabolic pathways;<br>Pyrimidine metabolism
2-Hydroxyethanesulfonate	map00430; map01100	Metabolic pathways;<br>Taurine and hypotaurine metabolism
2'-Deoxyinosine	map00230; map01100; map01232; map02010	ABC transporters;<br>Metabolic pathways;<br>Nucleotide metabolism;<br>Purine metabolism
Phenyllactic acid	map00270; map01100	Cysteine and methionine metabolism;<br>Metabolic pathways
N1-Methyl-2-pyridone-5-carboxamide	map00362; map00621; map00627; map01100; map01110; map01120; map01220	Aminobenzoate degradation;<br>Benzoate degradation;<br>Biosynthesis of secondary metabolites;<br>Degradation of aromatic compounds;<br>Dioxin degradation;<br>Metabolic pathways;<br>Microbial metabolism in diverse environments
Adipic acid	map00230; map01100; map01232; map02010	ABC transporters;<br>Metabolic pathways;<br>Nucleotide metabolism;<br>Purine metabolism
2,6-Dichloroindophenol	map00260; map00270; map00300; map00920; map01100; map01110; map01120; map01230	Biosynthesis of amino acids;<br>Biosynthesis of secondary metabolites;<br>Cysteine and methionine metabolism;<br>Glycine, serine and threonine metabolism;<br>Lysine biosynthesis;

		Metabolic pathways; Microbial metabolism in diverse e nvironments; Sulfur metabolism
Alanyl-Isoleucine	map00240; map01100; map01232; map02010	ABC transporters; Metabolic pathways; Nucleotide metabolism; Pyrimidine metabolism
Coumarin	map00061; map00240; map00410; map01100; map01212	Fatty acid biosynthesis; Fatty acid metabolism; Metabolic pathways; Pyrimidine metabolism; beta-Alanine metabolism
Formyl phosphate	map00230; map01100; map01232; map02010	ABC transporters; Metabolic pathways; Nucleotide metabolism; Purine metabolism
H-LEU-VAL-OH	map00380; map01100	Metabolic pathways; Tryptophan metabolism
Isoformononetin	map00470; map00550; map01100; map01502	Amino acid metabolism; Metabolic pathways; Peptidoglycan biosynthesis; Vancomycin resistance
Isoleucyl-Leucine		
Isoleucyl-Phenylalani ne		
L-3,4-Dihydroxybutan -2-one 4-phosphate		
Methylphosphote		
Nitroprusside		
O-Phosphorylhydroxy lamine		
Tetrafluoroethylene		
**Top30_Best_Name**		
D(+)-Glucose		
L-Aspartic acid		
5'-Methylthioadenosin e		
Benzoic acid		
Adenosine		
L-Homoserine		
Uridine		
Malonic acid		
